# Monthly Summary

**Published:** 1868-12

**Authors:** 


### MONTHLY SUMMARY.

*Hypertrophy of the Right-half of the Body in a Child aged four years, first noticed shortly after Birth.*—By Samuel Logan, M.D., Professor of Surgery, New Orleans School of Medicine.

O. B., Daughter of Mr. T. B., at present a resident of Brashear City, aged 4 years; well grown for age—parents, as well as self, are natives of Louisiana; has seven brothers and sisters, all of whom are healthy. The parents are both healthy in every respect; the mother has born twins three times, the child who is the subject of these notes not being, however, one of the twins. The family history as regards health is excellent, except that one of the mothers' brothers has two out of six children deaf and dumb, but the mother of these children is delicate and in no way related by blood to the family of this child.

About ten days after birth, the right-half of the person of the infant was found to be larger than the other, and since that time the disproportion has seemed steadily to increase. She is now a strong hearty child; but the disproportion is marked in every particular, more so, however, when the opposite extremities are compared. One leg is about an inch larger than the other causing a peculiar gait, and the foot requires a shoe of at least one and a half or two sizes larger, and the two arms and hands show a corresponding disproportion. The difference is, of course, not so marked, but can be readily seen, in the head, the eyes, the nose, the tongue and the trunk all the way down. The only very serious attack of sickness from which the child has ever suffered she experienced in September, 1867, when she was very ill with Yellow Fever having had black hæmorrhage by the bowels. I am informed that her recovery from this attack was tedious, and that about ten days after convalescence was cosidered established, a portion of the alveolar process of the lower left maxillary containing one incisor and a canine tooth, the latter embryonic, sloughed off. Since this she had been restored to her usual good health. Deeming the case a remarkable one, never in fact having heard of a similar, I took a few notes embodying the above facts, and beg leave to report them without comments to the Association.—*N. O. Jour. of Med.*

---

*Parasite of the Cheek.*—Dr. G. H. Vance in the *Med. and Surg. Reporter* relates the following case.

In the month of February, 1867, while residing in Victoria, Illinois, a boy, five years of age, and of a scrofulous diathesis, came into my office, accompanied by his mother, who wished me to examine the little fellow's left cheek. There was quite a protuberance manifested, in character approaching that of a good-sized boil, which was attended with redness, and at times considerable pain, causing irritableness of temper, sleeplessness, and anorexia. Thinking it arose from an impoverished state of the system, I prescribed an alterative cathartic, with a local application of ung. hydg. nit. But at the expiration of a week, contrary to my diagnosis, and much to my astonishment, upon slight pressure, a curious object was developed, which upon examination, proved to be alive, three-fourth of an inch in length nearly transparent, and surrounded with several rings or joints. Upon removal of the object, the tumefaction subsided, and the cheek soon returned to its natural appearance. As I was in possession of no microscope, my parasitic investigations were very much limited and unsatisfactory. No case of the kind has since appeared in any member of the family, and as far as I could ascertain, (within bounds of possibility,) the patient had eaten no raw meat or vegetables. Therefore, what caused the appearance of the animal in this particular? How was the germ sown? Was it a species of guinea worm, trichina spiralis, acarus scabii, or what?"

---

*Carbolic Acid in Burns.*—Dr. E. R. Squibb states (*Med. Gazette*) that "some of the applications of carbolic acid are really but revivals of old methods of practice; thus creosote has a very deservedly high reputation as an application to burns, and as carbolic acid is medicinally at least identical with creosote, it is but changing the name of the agent. In my own person I have experienced its benefits, and once tried the following experiment;

Accidentally scalded by a jet of steam, a solution of carbolic acid which is kept constantly on hand for such purposes. was at once applied on a piece of lint, before the pain became severe. In two or three minutes the pain was gone. In an hour the dressing was removed and the pain then returned. The dressing was renewed and the pain again ceased, but to return again when the dressing was removed. This anodyne effect has not been explained as yet and does not appertain to its peculiar character as an antizymotic. A boy employed in my factory broke a bottle of compound spirits of ether, saturating the front of his pantaloons ; this then took fire from a lamp which he was using, and he was quite severely burned over the lower part of the abdomen, the genitals, and the upper part of the thighs. In my absence he was taken to his home and dressed with cotton and oil. In a few hours I obtained permission from his medical attendant to apply the carbolic acid, which was attended with speedy relief of his pain and he had a quick recovery. This application of the carbolic acid is one of its most valuable uses."

---

*Styptic Paper.*—It is well known that the perchloride, or as it is some times called, the muriate of iron possesses styptic or hæmostatic properties in a high degree. To obviate the inconvenience of carrying it about in solution, and also of applying it, M. Gustave Gabillon, of Paris, has devised a "styptic pape," the method of preparing which is as follows :

In a vessel carefully tinned inside, he keeps boiling for four hours, (skimming from time to time, and adding water to replace that evaporated,) a solution of one pound of best quality gum benzoin, one pound of rock alum, and four and a third gallons of water. As soon as the solution is cooled it is to be filtered, and the paper or tissue te be dipped into it, kept there until saturated, and then carefully dried, after which a solution of the perchloride, more or less concentrated, is applied with a brush or roller. The paper or tissue thus prepared may be preserved for any length of time in a state always ready for use, by excluding it from air.—*Exchange.*

---

*Liquefying Laughing Gas*—The uniform efficiency and safety of laughing gas as an anæsthetic, has prompted *The British Medical Journal* to suggest that a bottle be made strong enough to hold the gas in a liquid form, and of such weight and dimensions that it may be easily carried by the surgeon in his daily rounds. At present it is used by dentists from large gas-bags, into which it is placed as soon as made. Laughing gas is composed, according to the new notation, of two atoms of nitrogen and one of oxygen. These two elements are the principal constituents of common air. Laughing gas or nitrous oxyde can be liquefyed under a pressure of 750 pounds per square inch when at the temperature of 45° Fahr. The most convenient and safe receptacle for the liquid would be a brass or copper tube, not more than a foot in length, and of such thickness as to resist a pressure of at least 1,500 pounds, or several small tubes of the ordinary thickness might be united side by side and made entirely safe.

To get the nitrous oxyde gas into a portable shape, is certainly "a consummation devoutly to be wished."—*Med. and Surg. Reporter.*

---

*Division and Excision of Nerves.*—Some months since, a case occurred in Paris, which attracted much attention, and caused considerable speculation. A woman received a wound just above the wrist, which completely divided the median nerve, notwithstanding which the peripheral end of the nerve was painful when touched, and the fingers retained the property of feeling. Some observers sought to explain the retention of the sensibility by supposing an anomalous distribution of the nerves, but Professor Richet believed the painful impression produced by touching the peripheral extremity of the nerve to be due to the presence of *nervi nervorum,* lately discovered by M. Sappey; and the retention of sensibility by the fingers was in consequence of the corpuscles of tact being supplied by filaments from the radial and ulnar nerves, as well as the median, as demonstrated by Cruveilhier and Robin. In consequence of the interest manifested in the above case Dr. J, F. Miner has been induced to record in the June number of the *Buffalo Medical and Surgical Journal,* three others of a similar nature, which have occurred in his practice. A man received a gun-shot wound of the arm, the ball passing directly through the median nerve. False anchylosis eventually ensued, and the pain from the receipt of the injury was intense and indescribable. Twenty-two weeks from the time of injury three inches of the nerve, which was found closely involved in the cicatrix, were removed, the pain ceasing immediately. *Sensation* was at no time lost, but was at first somewhat diminished along the anterior portion of the forearm and hand.—The second case was one of cystic tumor of the arm, which had existed five years, latterly becoming exceedingly painful. It so involved the musculo-spiral nerve that the removal of the tumor necessitated the removal of a portion of the nerve. This was followed by loss of motion, and in great degree of sensation, of all the parts supplied by the nerve. In the third case, a wound dividing all the flexor tendons of the middle, ring, and little fingers, the ulnar artery and nerve, *sensation* was almost entirely lost immediately after the injury, but was afterwards perfectly regained, the wound healing rapidly and the nerve, of course, uniting.—*Chicago Med. Exam.*

---

*The Injurious Consequences of the Use of Sewing Machines Prevented.* *Mr. Editor*,—Some time since, in an article published in the Medical and Surgical Journal (See this Journal, Vol. lxxv. page 87,) we called the attention of its readers to the important subject of the bad effects often produced on the health of females by the frequent and prolonged use of sewing machines. In that article a translation was given of a portion of a paper on the same subject read to the *Societe Medicale des Hopitaux*, of Paris, by Dr. Guibout. Subsequent experience has confirmed us in the opinion that much harm is done by these instruments solely for the want of some proper motive power by which the operator may be relieved from the excessive labor of working the treadle; and in this opinion we believe we are sustained by most physicians. Our object in writing at this moment is to say, that there seems to be a prospect that this objection to this otherwise invaluable machine, will be entirely removed by an ingenious invention just patented by Dr. Spencer, a dentist of Providence. This contrivance he calls an "improved mode of producing a rotary motion from the treadle;" and the effect of it is that the motion is kept up by the slightest movement of the foot. In the case of ordinary treadles acting upon a crank, the foot, of necessity, must move, with each revolution, through the same distance up and down; and the effect of this monotonous repetition of the movement is most wearisome and exhausting. By Dr. Spencer's contrivance the machine is kept constantly in action whether the foot moves through a longer or shorter distance; giving the operator a chance of varying, as often as is desired, the muscular effort necessary to run it. The invention displays great ingenuity, and attracted much attention at the last meeting of the Society of Arts at the Institute of Technology. So far as it has been tested it has proved to be all that its inventor claims for it. S.L. ABBOT.—*Boston Med. and Surg. Journal.*

---

*Curious Exostotic Growth*—Dr. Hutchinson presented to the New York Pathological Society the malar bone, with the corresponding half of the inferior maxilla, removed from a patient of the Brooklyn City Hospital, who died of dysentery. While under treatment for this disease, his attending physician, Dr. Crane, called the attention of Dr. Hutchinson to the existence of anchylosis of the jaw On examination, a bridge of bone was discovered to extend from the malar bone to the inferor maxilla, following the anterior margin of the masseter muscle. An operation for his relief was devised, and would have been performed had he recovered from his dysentery. At the *post-mortem* examination, an exostotic growth was found to take its origin in the malar bone, and extend to the maxilla, being connected with the latter by a firm ligamentous attachment. The articulation of that side of the jaw remained healthy. This growth of bone was attributed by the patient to a fall upon the side of his face some years before, while on shipboard.—*Medical Record.*

---

*A Good Cement*, which may be rendered sufficiently flexible by the addition of a little glycerine, is made by digesting one ounce of finely cut isinglass (true) and two of gelatine in a quart of water, straining the mixture, and evaporating it to six fluid ounces; these are then mixed up with a scruple of mastich dissolved in half an ounce of alcohol and rubbed up with a little zinc-white and varnish.—Or, gutta-percha in shreds (one or two parts) is dissolved by agitating with (10 parts of) oil of turpentine, and the solution, when strained and warmed, is mixed with (10 parts of) linseed-oil varnish.—*Chem. Gazette.*

*Anæsthesia and the Mode of Action of Anæsthetics.*—Dr. Sansom considers that it is particularly important now that much attention is directed to practical anæsthesia, to determine the *rationale* of action of anæsthetics. He criticized the theory of their direct action on the central ganglia of sensation, which he considered disproved by many facts. On the contrary, they presented in their union a complete similarity with the phenomena of deprivation of oxygen. Tracing the action of a typical anæsthetic, the author showed that it acted both as a cardiac and vaso-motor stimulant: it contracted the systemic arteries, producing a condition of insufficient supply of arterial blood. Dr. Sansom entered into a critical examination of the analogies and correlations of this condition, showing, first, an indentity with cold and with galvanism, which by a similar action produced similar results; he traced the effect upon the capillary circulation, showing that the velocity of the latter was diminished, but the blood was expressed towards the venous channels in which it accumulated, hence the distension of the right side of the heart witnessed in *post-mortems*. Chloroform, however, when its vapor is insufficiently diluted with air, has the power of superinducing paralysis of the cardiac and vaso-motor forces—hence its danger. Anæsthetics act on the blood-corpuscle, especially in the hæmoglobin, impeding its oxygenation. The author concluded that anæsthetics produce these phenomena by inducing a suppression of oxidation in the body, 1. directly by acting on the blood; 2, indirectly, by modifying the forces by which the blood is circulated; and that they have no special action on sensory ganglia.—*Proceed. Brit. Med. Assoc., Brit. Med. Journal.*

---

*Diagnosis of Cancrum Oris.*—At the Dublin Hospital (*Med. Press and Circ.*) Mr. Croly one of the hospital surgeons, made some remarks in reference to the diagnosis of this disease, after an operation made on the person of a little child five years of age. He stated that the cancrum oris is a curious sequela of the measles, and found among delicate and ill-nourished children. This disease is often confounded with mercurial salivation, but may easily be diagnosed by an account of the case, and by the fact of this disease attacking only *one* side of the mouth, while mercurial sloughing occurs at both sides.—*Medical Record.*

